# Supplementary material for: Collaborative intelligence in AI: Evaluating the performance of a council of AIs on the USMLE
Source: PLOS Digit Health. 2025 Oct 9;4(10):e0000787. doi: 10.1371/journal.pdig.0000787 (PMC12510544; doi:10.1371/journal.pdig.0000787)
Supplement: S2 File — (ZIP) [file pdig.0000787.s002.zip › S2 Data Raw data files containing unprocessed question inputs and LLM outputs including transcripts of deliberations/STEP3_Question-111-1_Type-MC-NJ.docx]

**Facilitator** **[Round 1]:** A 7-year-old boy with sickle cell disease is brought to the office by his father because of fever and bone pain. The father says that his son was discharged 5 days ago after a 3-day hospitalization for the same symptoms. The patient had been admitted with a diagnosis of vaso-occlusive crisis with bone infarct, with resolution of symptoms after treatment with intravenous fluids, narcotics, and antibiotic therapy for 3 days. Blood cultures were negative. The patient's back pain and fever recurred 2 days ago and seem more severe than prior to the hospitalization. His medical chart shows that he had one other uncomplicated hospital admission at age 4 years for a vaso-occlusive crisis with lower extremity bone pain. Vital signs today are temperature 39.2°C (102.5°F), pulse 110/min, respirations 24/min, and blood pressure 115/60 mm Hg. The patient is uncomfortable but does not appear toxic. Cardiac examination discloses a grade 2/6 systolic ejection murmur at the left lower sternal border. Spleen is not palpated. There is considerable tenderness over the L1–L3 region of the back without fluctuation. There is diffuse pain in both thighs without localization, but range of motion is normal. MRI of the spine confirms the diagnosis. Which of the following is the most appropriate initial intravenous pharmacotherapy? (A) Ceftriaxone (B) Cephalothin and clarithromycin (C) Clindamycin and gentamicin (D) Nafcillin (E) Vancomycin and cefotaxime

**AI_E:** RESTATING THE QUESTION: So, we are dealing with a 7-year-old boy who has a history of sickle cell disease and has been experiencing a recurrent fever and bone pain. Despite a short hospital stay where he received fluids, pain control, and antibiotics, the boy's symptoms have not only returned but appear more severe. The clinical findings of bone tenderness and the MRI confirmation suggest that we are dealing with another episode of vaso-occlusive crisis with bone involvement, most likely a bone infection or osteomyelitis. The objective here is to establish the most effective initial intravenous (IV) pharmacotherapy from the given choices. PLANNING: Assumptions that go into making this choice would include which bacteria are most likely responsible for the infection, the patient's age, his underlying disease, and the localization of his symptoms. Sickle cell patients are particularly susceptible to certain pathogens, notably encapsulated bacteria due to their functional asplenia. Salmonella species, for instance, are often responsible for osteomyelitis in these patients. Evaluating this assumption might involve considering his previous response to antibiotic therapy, the lack of identified pathogen from his blood culture, and the recurrent nature of his condition. CLINICAL REASONING: Despite results from the blood cultures being negative, infection is still a prime suspect given the patient's history, symptoms, and their recurrence. In sickle cell disease patients, vaso-occlusive crises often lead to bone infarcts which make bone tissue more susceptible to infections, mainly by encapsulated organisms such as Streptococcus pneumoniae and Salmonella species. The pattern of the boy's symptom recurrence after initial improvement with antibiotics and the severity of symptoms point heavily towards the presence of an infection, more specifically, osteomyelitis. This is due to the repeated pattern of vaso-occlusive crises leading to bone infarcts, which then become focal points for infection. CONCLUDING: Therefore, initial intravenous pharmacotherapy for this patient should ideally target the most commonly implicated organisms causing osteomyelitis in sickle cell disease. In this case, those would be Streptococcus pneumoniae and Salmonella species which are effectively treated with a combination of a third-generation cephalosporin, such as ceftriaxone or cefotaxime, and vancomycin. RESPONSE: Hence, the most appropriate initial intravenous pharmacotherapy for this patient would be (E) Vancomycin and cefotaxime.

**AI_D:** The question asks us to determine the most appropriate initial intravenous pharmacotherapy for a 7-year-old boy with sickle cell disease, who presents with fever and bone pain. He had been discharged 5 days ago following a hospitalization for a similar vaso-occlusive crisis with bone infarct. Despite treatment with fluids, narcotics, and antibiotics, his back pain and fever have recurred and seem more severe than before his hospitalization. It's clear that the patient's presenting condition is recurrent and has intensified since his hospitalization. To summarize, we're seeking for an effective intravenous pharmacotherapy given this history. Several assumptions could be made: (1) the previous antibiotic treatment might not have been effective thus a different kind of antibiotic therapy might be required; (2) the pain could be unrelated to an infection thus requiring a different management; and (3) the patient may be developing a condition associated with sickle cell disease that might need a different treatment approach. Given the symptoms and the recurrence after treatment, it's plausible to assume that the boy is dealing with a complication of sickle cell disease, more specifically, osteomyelitis, even though blood cultures were negative. Osteomyelitis is an infection of the bone that is common in sickle cell disease patients, with the most common organism being Salmonella followed by Staphylococcus. Fever and bone pain, as presented by the patient, would be symptoms of osteomyelitis, and the recurrence of symptoms after treatment suggests the initial treatment might not have been adequate to cover the organisms that commonly cause osteomyelitis in sickle cell patients. When considering the clinical reasoning, the prioritized alternate assumption leads us to look for an antibacterial that can effectively treat Salmonella and Staphylococcus. Ceftriaxone would be the most common antibiotic to cover these organisms. However, the continuation of symptoms despite previous treatment could indicate Salmonella osteomyelitis. While ceftriaxone has activity against Salmonella, it may not fully penetrate bone tissue, hence the need for an alternative therapy. The final response regarding the question about the most appropriate initial intravenous pharmacotherapy, given the symptoms and assumptions made, leads us to one choice: Vancomycin and cefotaxime (E), since this combination can provide broad coverage against Staphylococcus and has better bone penetration to effectively treat Salmonella osteomyelitis in children. Thus, the response, under this pattern of reasoning, would be (E) Vancomycin and cefotaxime.

**AI_B:** RESTATING THE QUESTION: The clinical scenario presents a 7-year-old boy suffering from sickle cell disease, who has been suffering from recurrent fever and bone pain, severe enough to warrant hospitalization. These symptoms are characteristic of a vaso-occlusive crisis with bone infarct, a common, severe complication of sickle cell disease in which the sickled red blood cells obstruct small blood vessels, leading to ischemia and infarction of the bone. Despite a previous treatment regimen that included intravenous fluids, narcotics, and antibiotics, the boy's symptoms have returned and increased in severity. The question asks which initial intravenous pharmacotherapy would be appropriate in this situation. PLANNING: Considering the patient's symptoms, previous disease course, and the timeline of his recent hospitalization, it is pertinent to consider the high likelihood of an infection playing a role in his symptom recurrence. Given that blood cultures were negative, we might speculate that the infection may be localized. Based on the patient's symptomatology, this could be a bacterial infection, potentially due to Streptococcus pneumoniae or Haemophilus influenzae, both encapsulated organisms known to pose significant risk to individuals with sickle cell disease, as their splenic dysfunction impairs their ability to mount an effective immune response against these bacteria. CLINICAL REASONING: In such patients, the additional stress of an infection could trigger a vaso-occlusive crisis and exacerbate pre-existing symptoms. Therefore, attentive supportive care, robust hydration, analgesics for bone pain, and empiric antibiotic therapy targeting potential invading organisms should be the mainstay of initial management while cultures are obtained and monitored. Treatment should preferably be broad-spectrum, effective against the organisms most likely to be encountered in this clinical context. CONCLUDING: In light of the above, with the patient's symptoms and pathology, the most appropriate initial intravenous pharmacotherapy would be a regimen that provides the broadest coverage against the organisms most likely to be implicated in this clinical scenario. RESPONSE: The most appropriate initial intravenous pharmacotherapy would be (E) Vancomycin and cefotaxime. These drugs provide broad coverage against both Gram-positive and Gram-negative bacteria, including the aforementioned Streptococcus pneumoniae and Haemophilus influenzae, and therefore gives the highest likelihood of tackling any potential infection that may be exacerbating this patient's vaso-occlusive crisis.

**AI_A:** RESTATING THE QUESTION: The query seeks to identify the most suitable intravenous pharmacotherapy for a 7-year-old boy afflicted with sickle cell disease who is displaying symptoms of fever and bone pain, as well as a recent recurrence of these symptoms post-hospitalization. The boy's diagnosis has been confirmed via MRI, and his medical history includes vaso-occlusive crises causing bone infarcts. The symptoms are more severe now than prior to his previous hospital admission. His physical examination and vital signs have been indicated, including a systolic ejection murmur. PLANNING: The crucial point here is the recurrent bone pain and fever after hospitalization. This recurrent pyrexia and bone pain are suggestive of an underlying septic condition, perhaps osteomyelitis. Among patients with sickle cell disease, one bacteria, Salmonella, is particularly invasive, capitalizing on the compromised blood supply to the infarcted bone, where the patient's immune defenses are weakened. Hence, empiric treatment should target gram-negative enteric organisms and Staphylococcus aureus, the most common pathogens for osteomyelitis in patients with sickle cell disease. CLINICAL REASONING: In patients with sickle cell disease who develop an infectious crisis, infection with encapsulated organisms such as Pneumococcus, Haemophilus influenzae, and Salmonella should always be at the top of the differential diagnosis. However, a vaso-occlusive crisis (VOC) can mask infection, as the symptoms—namely fever and pain—are typical in both scenarios. Given the patient's recent hospitalization, a recurrence of symptoms post-discharge gives a higher suspicion of an infectious process rather than a simple VOC. Moreover, negative blood cultures don't rule out osteomyelitis; many cases of osteomyelitis can have negative blood cultures. CONCLUDING: Given the most likely diagnosis of osteomyelitis, the choice of antibiotics should reflect the need to cover both gram-positive organisms (Staph aureus) and gram-negative organisms (Salmonella). This recommendation is supported by the boy's recurrent symptoms and MRI findings. RESPONSE: Therefore, the most appropriate initial intravenous pharmacotherapy is (E) Vancomycin and cefotaxime. Vancomycin is used to cover Staphylococcus aureus, and cefotaxime targets gram-negative bacteria including Salmonella — both primary culprits of osteomyelitis in sickle cell patients.

**AI_C:** RESTATING THE QUESTION: The present case revolves around a 7-year-old boy suffering from sickle cell disease. The boy has been experiencing recurring symptoms of fever and bone pain, originally diagnosed as a vaso-occlusive crisis with bone infarct. The symptoms have re-emerged, more severe than before, even after treatment, indicating another similar crisis or a different complication. Upon physical examination, the patient does not appear to be in a toxic state, with key (patho)physiological indicators being considerable back pain, diffuse thigh pain despite normal range of motion and a systolic ejection murmur found within the cardiac examination. We are required to determine the most appropriate initial intravenous pharmacotherapy to alleviate the patient's suffering. The options presented are Ceftriaxone, Cephalothin and clarithromycin, Clindamycin and gentamicin, Nafcillin, or Vancomycin and cefotaxime. PLANNING: From the symptoms and the previous hospital admission, it's not wrong to infer that the boy is experiencing another vaso-occlusive crisis. However, due to the increased severity and fast recurrence after treatment, it's feasible to hypothesize that the patient may have developed a more severe complication such as osteomyelitis. Also, the fact that the blood cultures were negative during his previous hospitalization, yet the symptoms returned, indicates there might be a micro-organism that was not initially captured. We should consider whether this could be a hospital-acquired infection or community-acquired infection given the course of illness. CLINICAL REASONING: The occurrence of vaso-occlusive crisis can predispose a patient with sickle cell disease to develop osteomyelitis due to infarction and subsequent sequestration, making the bone more prone to infections. The commonest organism responsible for osteomyelitis in sickle cell patients is Salmonella species followed by Staphylococcus aureus. Thus, an ideal initial intravenous pharmacotherapy should cover both these organisms effectively. CONCLUDING: I would suggest empiric antibiotic therapy which has a broad-spectrum, covering both Gram-positive and Gram-negative bacteria, more specifically Staphylococcus aureus and Salmonella species. The choice of antibiotic(s) should also be safe for pediatric use since the patient is a 7-year-old boy. RESPONSE: Given the clinical scenario and the need to cover both Staphylococcus and Salmonella, the combination therapy of (E) Vancomycin and cefotaxime would be the most optimal choice. Vancomycin is a glycopeptide antibiotic that is very effective against Gram-positive cocci, including Staphylococcus aureus. Cefotaxime is a third-generation cephalosporin which is effective against a wide variety of Gram-negative bacilli, including Salmonella. Both drugs can be used safely in pediatric patients.

**Facilitator** The consensus statement synthesizes the responses of AI_A, AI_B, AI_C, AI_D, and AI_E as follows: The case presents a 7-year-old boy with sickle cell disease experiencing recurrent fever and bone pain, symptoms characteristic of a vaso-occlusive crisis with bone infarct. Despite previous treatment with intravenous fluids, narcotics, and antibiotics, the symptoms have returned and increased in severity. The recurrence and intensification of symptoms post-hospitalization suggest a possible underlying septic condition, likely osteomyelitis. In patients with sickle cell disease, vaso-occlusive crises often lead to bone infarcts, making the bone tissue more susceptible to infections. The most common pathogens for osteomyelitis in patients with sickle cell disease are Salmonella and Staphylococcus aureus. Despite negative blood cultures, the possibility of osteomyelitis cannot be ruled out as many cases can have negative blood cultures. Given the clinical scenario, the most appropriate initial intravenous pharmacotherapy should cover both gram-positive organisms (Staph aureus) and gram-negative organisms (Salmonella). Therefore, the consensus among the AI responses is that the most suitable initial intravenous pharmacotherapy for this patient is (E) Vancomycin and cefotaxime. Vancomycin is effective against Staphylococcus aureus, and cefotaxime targets gram-negative bacteria including Salmonella, providing broad coverage against the organisms most likely to be implicated in this clinical scenario.
